# Phaeohyphomycosis in Transplant Patients

**DOI:** 10.3390/jof2010002

**Published:** 2015-12-22

**Authors:** Sanjay G. Revankar

**Affiliations:** 1 Division of Infectious Diseases, Department of Medicine, Wayne State University, 3990 John R. Street, 5 Hudson, Detroit, MI 48201, USA; 2 Harper University Hospital, 3990 John R., 5 Hudson, Detroit, MI 48201, USA; srevankar@med.wayne.edu; Tel.: +1-313-745-8599; Fax: +1-313-993-0302

**Keywords:** phaeohyphomycosis, voriconazole, itraconazole, posaconazole, transplantation

## Abstract

Phaeohyphomycosis is caused by a large, heterogenous group of darkly pigmented fungi. The presence of melanin in their cell walls is characteristic, and is likely an important virulence factor. These infections are being increasingly seen in a variety of clinical syndromes in both immunocompromised and normal individuals. Transplant patients are especially at risk due their prolonged immunosuppression. There are no specific diagnostic tests for these fungi, though the Fontana-Masson stain is relatively specific in tissue. They are generally seen in a worldwide distribution, though a few species are only found in specific geographic regions. Management of these infections is not standardized due to lack of clinical trials, though recommendations are available based on clinical experience from case reports and series and animal models. Superficial infections may be treated without systemic therapy. Central nervous system infections are unique in that they often affect otherwise normal individuals, and are difficult to treat. Disseminated infections carry a high mortality despite aggressive therapy, usually with multiple antifungal drugs. Considerable work is needed to determine optimal diagnostic and treatment strategies for these infections.

## 1. Introduction

Phaeohyphomycosis is caused by a large, heterogenous group of dematiaceous, or darkly pigmented fungi. Although rare, these infections are being increasingly seen in a variety of clinical syndromes in both immunocompromised and normal individuals. Transplant patients are especially at risk due their prolonged immunosuppression. In the Transnet database, phaeohyphomycosis accounted for 2.6% of all fungal infections seen, and were fairly evenly divided between stem cell (SCT) and solid organ transplant (SOT) patients [1]. These are difficult to classify into a simple framework and represent dozens of unique organisms [2,3]. As the number of patients immunocompromised from diseases and medical therapy increases, additional species are being reported as causes of human disease, expanding a long list of potential pathogens. As many of these are rarely seen clinically, referral to a mycology reference laboratory may be needed to accurately identify isolates to species level.

## 2. Epidemiology

Dematiaceous fungi are generally found in soil or associated with plants and distributed worldwide. Surveys of outdoor air for fungal spores routinely observe these molds [4]. Some studies have suggested these infections are more common in warmer climates [1]. Occasionally, species appear to be geographically restricted, such as *Rhinocladiella* (*R.*) (formerly *Ramichloridium*) *mackenzei*, which has primarily been seen in patients from the Middle East [5]. Exposure is thought to be from inhalation or minor trauma, which may not even be noticed by the patient. Since these are widespread in the environment, individuals are constantly exposed to them, though they rarely cause disease.

Most life-threatening infections due to these unusual fungi are seen in immunocompromised patients, with the possible exception of central nervous system (CNS) infection. However, infections due to certain species, such as *Lomentospora* (*L.*) *prolificans* (previously *Scedosporium prolificans*), have an extremely high mortality in immunocompromised patients despite aggressive therapy [6,7]. Fungal infections after SOT are uncommon, though associated with high mortality, ranging from 27% to 40%. The highest risk is seen in small bowel transplants (11.6%), followed by lung (8.6%), and liver (4.7%), with renal transplants having the lowest risk (1.3%) [8,9]. SCT patients are more likely to have disseminated disease [6,7].

## 3. Laboratory Diagnosis

Currently, the diagnosis of phaeohyphomycosis relies on pathologic examination of clinical specimens and careful gross and microscopic examination of cultures, occasionally requiring the expertise of a mycology reference laboratory for unusual or newly described pathogens. The Fontana-Masson stain, which is specific for melanin, can usually be used to confirm the presence of dematiaceous hyphae [10]. Unlike other more common fungal infections, there are no simple diagnostic tests to identify these fungi, particularly to the species level. No specific serologic, antigen or polymerase chain reaction (PCR) methods are routinely available, which is at least partly due to the tremendous diversity of these pathogens. Use of antigen based tests have not been very useful, with variable results. Antigen tests primarily used for Aspergillus and Candida, such as galactomannan and β-d-glucan, occasionally may be cross-reactive with this group of fungi, but this is not consistent [11,12]. A recent study examined the utility of bronchoalveolar lavage β-glucan levels, and found that BAL measurements were no better than serum levels in diagnosis [13]. However, studies have begun to examine the potential of identifying species within this group of fungi using PCR of highly conserved regions of ribosomal DNA [14]. Molecular identification has become more useful as one study demonstrated by identifying eight non-sporulating clinical isolates to the species or genus level by a combination of ITS and D1/D2 typing [15].

## 4. Pathogenesis

Relatively little is known regarding the pathogenic mechanisms by which many of these fungi cause disease. Invasive disease is uncommon in immunocompetent individuals; most are considered opportunists [2]. In dematiaceous fungi, one of the likely candidate virulence factors is the presence of melanin in the cell wall, which is common to all these fungi. Disruption of specific genes involved in melanin production leads to markedly reduced virulence in animal models [16,17]. There are several mechanisms proposed by which melanin may act as a virulence factor including scavenging free radicals and hypochlorite that are produced by phagocytic cells in their oxidative burst that would normally kill most organisms [18]. More recently, specific loss of function gene mutations in the CARD9 protein have been associated with an increased risk of developing disseminated infection due to *Exophiala* species [19]. The two patients in the study were otherwise healthy adults with no other opportunistic infections. Additional studies are needed to further elucidate genetic susceptibility to these rare infections.

## 5. Clinical Manifestations

A variety of infectious syndromes can be seen with these fungi, from superficial infections such as keratitis and subcutaneous nodules to invasive infections such as brain abscess and disseminated disease [20] (Table 1). In particular, phaeohyphomycosis encompasses many clinical syndromes due to a wide variety of dematiaceous fungi [2,10]. Clinical presentation may be indolent, particularly in solid organ transplant patients. At one institution, over 20 years, the incidence of phaeohyphomycosis in SOT patients was 0.7%, with an average time to diagnosis of 20 months from transplantation. Most were cutaneous infections, with an overall mortality of 19% [21].

**Table 1:** Clinical syndromes associated with phaeohyphomycosis and suggested therapy.

Clinical Syndrome	Commonly Associated Fungal Genera	Therapy
Subcutaneous Nodules	*Exophiala*	Surgery ± voriconazole
	*Alternaria*	-
	*Phialophora*	-
Keratitis	*Curvularia*	Topical natamycin ±
	*Bipolaris*	voriconazole
Pneumonia	*Ochroconis*	Voriconazole
	*Exophiala*	(Lipid amphotericin B if severe)
	*Chaetomium*	-
Brain abscess	*Cladophialophora* (*C. bantiana*)	Voriconazole or posaconazole ±
	*Rhinocladiella* (*R. mackenzei*) *Ochroconis*	lipid amphotericin B ± flucytosine-(optimal therapy unknown)
Disseminated disease	*Lomentospora* (*L. prolificans*)	Lipid amphotericin B
	*Bipolaris*	+ voriconazole + echinocandin
	*Wangiella*	(optimal therapy unknown)

In general, there are no standardized therapies for infections caused by these uncommon fungi. While antifungal susceptibility testing is standardized, including for some dematiaceous fungi, there are no interpretive breakpoints for any drugs, as clinical correlation data are lacking. The triazoles, itraconazole, voriconazole, posaconazole all have excellent activity against dematiaceous fungi, except for *Lomentospora prolificans*. Voriconazole and posaconazole are likely better tolerated than itraconazole with a broader spectrum of activity and would be preferred drugs. Isavuconazole is a novel broad-spectrum azole with tolerability profile comparable to fluconazole and less drug interactions than voriconazole and itraconazole [22]. Limited *in vitro* susceptibility data are available for dematiaceous fungi, though no clinical data has been published to date [23].

Both voriconazole and posaconazole are availability in IV formulations for seriously ill patients unable to tolerate oral therapy. Length of therapy is generally based on clinical response, and ranges from several weeks to several months or longer, including possible lifelong suppressive therapy in immunocompromised patients with infections such as brain abscess or endocarditis due to refractory pathogens. The diversity of the pathogens and of the hosts makes it impossible to find a “one size fits all” therapeutic strategy. Though no randomized trials have been done, these infections have become important enough to warrant guidelines published by the ESCMID that review disease states and make suggestions for diagnosis and management of specific clinical entities due to certain common species of dematiaceous fungi [24,25].

### 5.1. Superficial and Deep Local Infections

Superficial infections are the most common form of infection due to these fungi. Cases are generally associated with minor trauma or other environmental exposure. Although they rarely lead to life-threatening disease, significant morbidity can occur depending on the site of infection and response to therapy.

There are numerous case reports in transplant patients of subcutaneous infection due to a wide variety of species [25,26]. Minor trauma is the usual inciting factor, though it may be unrecognized by the patient. Lesions typically occur on exposed areas of the body and often appear as isolated cystic or papular lesions. Surgical excision alone has been successful in a number of cases [27]. Oral systemic therapy with an azole in conjunction with surgery is also frequently employed and has been used successfully, especially in immunocompromised patients [26,28].

Keratitis is an uncommon, but serious infection due to these fungi. Dematiaceous fungi account for up to 8%–17% of cases, particularly in tropical regions [29]. Topical polyenes, such as natamycin and amphotericin B, are commonly employed, but oral and topical itraconazole has been found to be useful as well, particularly in refractory cases [30]. Recent clinical experience suggests voriconazole and posaconazole are potentially useful agents as well [31,32]. Penetrating keratoplasty should be considered in those patients failing initial therapy. However, many patients are left with residual visual deficits at the end of therapy [29].

Deep local infection such as septic arthritis and osteomyelitis are rare and often difficult to treat, frequently requiring both surgical and combination medical therapy [33,34].

### 5.2. Pneumonia

This usually occurs in immunocompromised patients, and may be due to a wide variety of species [35,36]. It is unclear what specific risk factors may contribute to pulmonary infection with these fungi, which are commonly found in the environment, though a study by Campos et al found an association between pre-transplant colonization and subsequent pneumonia in lung transplant patients [36]. Mortality rates are high in immunocompromised patients. Experience with voriconazole is relatively limited, but given its activity in pulmonary aspergillosis and its broad spectrum would favor it in this setting as well [37]. Posaconazole was successfully used to treat a renal transplant patient with *Phaeoacremonium* pneumonia over 4 months [38].

### 5.3. Central Nervous System Infection

Though this is a rare clinical manifestation of infection, it remains one of the most difficult to cure and mortality rates are high. In contrast to other clinical syndromes, this can often occur in immunocompetent individuals typically involving dematiaceous fungi [39]. The pathogenesis may be hematogenous spread from an initial, presumably subclinical pulmonary focus.

In a retrospective analysis of 101 reported cases due to dematiaceous fungi, over half occurred in immunocompetent patients, with *C. bantiana* the most common species isolated [39]. Brain abscess was the primary clinical manifestation in 87 cases. Overall results of therapy suggested that the combination of amphotericin B, flucytosine and itraconazole may be associated with improved survival, though it was not frequently used. Therapy varied widely depending on the case report. Complete excision of brain abscesses appeared to have better outcomes than aspiration or partial excision. Outcomes were poor, with an overall mortality >70%.

Voriconazole was not used in the above case series but has been used in subsequent reports of *C. bantiana* brain abscess [40,41,42]. Only one of the cases, in an immunocompetent patient, was successfully treated [41]. However, clinical improvement was seen in one of the severely immunosuppressed patients while receiving voriconazole, despite later succumbing to the infection [42]. Posaconazole has also been used successfully, including a case of *Rhinocladiella* and *Scedosporium* brain abscess [43].

### 5.4. Disseminated Infection

This is the most uncommon manifestation of infection seen with filamentous fungi. In a recent review of dematiaceous fungi, most patients were immunocompromised, though occasional patients without known immunodeficiency or risk factors developed disseminated disease as well [6]. In contrast to most invasive mould infections, blood cultures were positive in over half the cases, usually seen with *L. prolificans* [6,7]. Infection with this species was also associated with septic shock. The mortality rate was >70%, despite aggressive antifungal therapy. There were no antifungal regimens associated with improved survival in disseminated infection, including multiple combination therapies [6,7].

Infection with *L. prolificans* was associated with nearly 100% mortality in the absence of recovery from neutropenia. A case of disseminated *E. spinifera* infection was treated successfully with posaconazole after failing itraconazole and amphotericin B [44]. Combination therapy with voriconazole and L-AmB was used to treat fungemia with *Exophiala dermatiditis* after failure of micafungin [45]. A retrospective study of 18 transplant patients by Husain et al suggested improved outcomes in *L. prolificans* infection treated with voriconazole compared to amphotericin B, though this was not statistically significant [37]. SCT patients were more likely to develop disseminated disease. Mortality rates remained high (>75%).

### 5.5. Alternative Therapy

There are few useful alternatives to triazole antifungal agents against these uncommon fungal infections. Amphotericin B may be useful for severe disease given its broad fungicidal activity against most pathogens, though isolates of *Scedosporium* are frequently resistant [46,47,48]. However, use of lipid AmB preparations allows for much higher doses than possible with standard AmB, which may improve their efficacy against these fungi. The pharmacodynamics of these formulations are different from standard AmB and may also affect their overall efficacy for specific infections [49]. However, given the need for parenteral administration, use of these agents is mostly confined to serious infections in unstable patients. Once the infection is under control, longer term therapy with a broad spectrum oral azole is often reasonable until complete response is achieved.

Terbinafine also inhibits ergosterol synthesis, but acts on a different target than azoles. It is also considered fungistatic, and its clinical role has been relegated to treatment of dermatophyte infections. However, *in vitro* activity is quite broad and includes many non-dermatophyte moulds [50]. There has been recent interest in potentially expanding its clinical spectrum [51,52]. However, its extensive binding to serum proteins and distribution into skin and adipose tissue have diminished enthusiasm for its use in treating serious systemic fungal infections [53]. However, terbinafine was used to successfully treat a case of *E. jeanselmei* subcutaneous infection in a heart transplant patient after failing itraconazole [54].

Combination therapy is a potentially useful strategy for refractory infections, though it has not been studied extensively and is not routinely recommended [55]. Terbinafine in particular appears to provide synergistic activity with azole antifungals, and this may be a useful strategy against refractory subcutaneous infections [56]. The mechanism is presumably potent inhibition of ergosterol synthesis at two different steps of the pathway by these agents. In addition, recent case reports have suggested that the combination of itraconazole or voriconazole with terbinafine may be synergistic against *L. prolificans* and improve outcomes [2,57,58]. This should be interpreted with caution, as terbinafine is not generally used for systemic infections, and these are anecdotal reports. No randomized trials have been done in this group of patients. However, in the setting of refractory infection with few therapeutic options, this strategy may be reasonable. Finally, a case of refractory bone and joint infection due to *L. prolificans* were treated effectively with the combination of voriconazole and caspofungin [59].

## 6. Conclusions

Phaeohyphomycosis, though rare, has become increasingly recognized in a wide variety of clinical syndromes, especially in immunocompromised patients. Transplant patients are at particular risk given their prolonged immunosuppression. Many species are associated with human infection, though relatively few are responsible for the majority of cases. As these are typically soil organisms and common laboratory contaminants, they may be regarded from clinical specimens as non-pathogenic. However, the clinical setting in which they are isolated should always be carefully considered before making decisions regarding therapy. Diagnosis depends on a high degree of clinical suspicion and appropriate pathologic and mycologic examination of clinical specimens. Therapy is evolving for many of the clinical syndromes described, and randomized clinical trials are unlikely given the sporadic nature of cases. Itraconazole, voriconazole and posaconazole demonstrate the most consistent *in vitro* activity against this group of fungi. Given the lack of comparative clinical data, however, decisions over which azole to use in a particular setting will largely be empiric, though voriconazole has accumulated considerable experience given its ease of use and broad activity. Much additional work is needed in order to optimize therapy for these often refractory infections.
